# A large-scale seismic risk assessment framework using enhanced FEMA P-58 and Bayesian network inference: a case study of District 2, Tehran

**DOI:** 10.1038/s41598-026-50024-0

**Published:** 2026-05-02

**Authors:** Reza Hajirostami, Mehdi Banazadeh

**Affiliations:** Department of Civil and Environmental Engineering, Amirkabir University of Technology, Tehran, Iran

**Keywords:** Large scale seismic risk assessment, Incremental dynamic analysis (IDA), Bayesian network, GIS mapping, Engineering, Natural hazards, Solid Earth sciences

## Abstract

Seismic risk assessment is a probabilistic approach that evaluates the likelihood of earthquake occurrence, structural response, expected damage levels, economic losses, and potential casualties by incorporating the inherent uncertainties associated with seismic hazards and urban building characteristics. The primary objective of this study is to quantify and spatially characterize the distribution of damage states at the urban scale. Buildings were classified according to their structural system, age, and number of stories. The structures were initially modeled, analyzed, and designed in ETABS, and the beam and column section properties were extracted for each structural type. Finite element models were subsequently developed in OpenSees, and Incremental Dynamic Analysis, IDA, was performed to evaluate the seismic performance of building groups and large-scale seismic risk. The application of this approach to urban-scale seismic risk evaluation distinguishes this research from similar previous investigations. Given the considerable number of models, the extensive dataset, and the necessity for updating results under varying input conditions, a Bayesian Probabilistic Network was employed. In addition, GIS-based mapping was used to present the findings, including the exceedance probabilities of different damage states and the spatial distribution of collapse probability. The outcomes of this study identify areas that may exhibit relatively higher seismic vulnerability, emphasizing the potential need for targeted retrofitting strategies or, enhanced preparedness for post-earthquake emergency response and rescue operations.

## Introduction

Urban-scale seismic risk assessment requires the evaluation of the seismic performance of all buildings within a city, together with consideration of lifeline systems and accessibility networks. This process involves substantial uncertainties, which inherently increase its complexity. It is practically impossible to account for all parameters that influence seismic risk at the urban scale, from uncertainties associated with earthquake occurrence to variability in structural characteristics and urban infrastructure conditions. However, the greater the availability of reliable data and the more comprehensively these parameters are incorporated into the modeling framework, while maintaining computational feasibility, the closer the results will approximate reality.

Several earlier studies have attempted to address this problem using different approaches. One prominent example is the Risk Assessment Tools for Diagnosis of Urban Areas against Seismic Disasters, RADIUS, project, which provided nine cities with tools to estimate earthquake damage and manage seismic risk. The RADIUS methodology employs the intensity of past earthquakes to estimate potential future damage. This intensity is measured using the Modified Mercalli Intensity, MMI, scale, which is correlated with a ground motion parameter known as Peak Ground Acceleration, PGA. To estimate PGA values, the project recommends the use of one of three established empirical relationships^1–3^. For building damage assessment, structures are categorized into ten common structural types that significantly influence seismic performance, and a damage estimation model is developed for each category. However, because RADIUS was designed to be relatively simple, it omits several critical parameters and uncertainties, including detailed earthquake source mechanisms, occurrence probabilities, variations in structural design, and local soil conditions. The exclusion of these influential factors may introduce potential inaccuracies into urban seismic risk assessment outcomes.

The Federal Emergency Management Agency, FEMA, developed the HAZUS platform as a standardized framework for estimating losses from earthquakes, hurricanes, and floods. HAZUS is a GIS-based system designed for application across the United States. It provides a consistent procedure for assessing earthquake hazards, estimating physical damage to buildings and infrastructure, and evaluating casualties and economic losses. The method employs spectral analysis to determine the maximum structural response during ground shaking. Building vulnerability is subsequently classified according to four predefined damage states. In a related study, Del Gaudio et al.^4^ evaluated the performance of reinforced concrete buildings during the L’Aquila earthquake. They developed analytical models to generate pushover curves for vulnerability assessment and defined damage levels according to the EMS-98 scale. Their methodology incorporated random variables and employed Monte Carlo simulation to account for the probability of structural failure. Similarly, Riga et al.^5^ adopted a different approach to assess earthquake risk at the urban scale. They applied the Capacity Spectrum Method to evaluate the seismic risk of Thessaloniki, Greece. Their study emphasized the quantification of uncertainty in large-scale urban risk assessment and provided a structured probabilistic framework for analysis. Boukri et al.^6^ conducted a city-wide earthquake risk assessment for buildings in Algeria. Their primary objective was to apply the RADIUS methodology to evaluate urban vulnerability and to estimate the associated economic losses and social impacts. Their findings demonstrated the practical applicability of structured, city-scale assessment methods for evaluating the potential consequences of earthquakes in rapidly developing urban areas. In another study, Bayraktarli et al.^7^ investigated the application of Bayesian probabilistic networks in earthquake risk assessment. They proposed a framework for risk management across three temporal phases, before, during, and after an earthquake. Using OpenSees, they modeled buildings and compared two categories, retrofitted and non-retrofitted structures. This work demonstrated the capability of probabilistic network models to support decision-making in large-scale urban risk management. In a comprehensive urban risk assessment, they integrated building data within a GIS environment and evaluated 7,186 reinforced concrete moment-resisting frame buildings, incorporating parameters such as building height, floor area, and local soil conditions. The dynamic properties of the buildings were estimated based on the procedures proposed by Priestley^8^ and the Turkish Seismic Code (1998)^9^. For retrofitted buildings, strength and displacement capacities were calculated using the methodology presented by Dazio^10^. Structural performance was evaluated using two primary indicators, maximum displacement and residual displacement. The Bayesian network was subsequently employed to determine the necessity of retrofitting based on the assessed risk levels.

Bayraktarli et al.^11^ applied Bayesian networks to assess earthquake risk in Adapazari, Turkey. They first evaluated the seismic hazard by considering sixteen different combinations of earthquake magnitude and source-to-site distance. For each combination, twenty synthetic ground motion records were generated. Buildings were classified according to the number of stories, year of construction, and occupancy type, such as residential or office use. Dynamic analyses were performed using OpenSees to evaluate structural response. From these analyses, the maximum interstory drift ratio, MIDR, was calculated. Three damage states were defined, and fragility curves were developed to represent the probability of reaching each damage state. Using GeNIe software, Bayesian probabilistic networks were employed to estimate the seismic risk for each individual building.

Previous studies that did not incorporate Bayesian networks generally relied on methodologies such as RADIUS, HAZUS, or RISK-E, the European platform for assessing urban risk. These approaches do not typically use nonlinear dynamic analysis to evaluate structural performance. Instead, damage assessment is commonly based on predefined empirical fragility curves derived from observational data. The application of Bayesian networks enables the incorporation of multiple sources of uncertainty, including those related to seismic hazard and urban-scale vulnerability. However, many studies have been limited to simplified representations, such as single-degree-of-freedom systems or basic damage models, primarily because nonlinear dynamic analysis is computationally demanding and detailed structural data at the city scale are often unavailable. Reliance on generalized damage models may therefore introduce inaccuracies into risk estimates.

The Federal Emergency Management Agency has established a comprehensive performance-based assessment framework in FEMA P-58 guidelines^12^. This methodology is fundamentally based on Incremental Dynamic Analysis, IDA, a systematic but computationally intensive procedure. The resulting IDA curves are subsequently used to derive building-specific fragility functions. Within the FEMA P-58 framework, building damage and associated economic losses are estimated while accounting for uncertainties in structural response and collapse fragility^13,14^. Moreover, the guidelines provide simplified procedures for estimating building response, which are particularly suitable for preliminary design stages and situations where full response history analysis is impractical^15^. The framework evaluates building-level losses by aggregating component-level damage, including both structural and nonstructural elements^16^. Nouri et al.^17^ applied the FEMA P-58 framework to assess the effectiveness of dampers in retrofitting deficient steel moment-resisting frames. Their analyses indicated that the inclusion of dampers increased structural stiffness, reduced yield drift, and prevented collapse under major earthquake intensity levels. Retrofitting also reduced repair time by nearly 50% under severe ground shaking, demonstrating that dampers represent a cost-effective seismic strengthening strategy. Bayesian Network, BN, inference provides a robust framework for modeling the complex and uncertain interdependencies inherent in seismic risk, linking variables such as earthquake intensity, soil liquefaction potential, and building damage^18,19^. By integrating outcomes from detailed FEMA P-58 performance assessments, Bayesian networks can incorporate critical uncertainties and conditional dependencies, thereby enhancing the accuracy and reliability of comprehensive risk evaluations^20^. Furthermore, Bayesian approaches facilitate iterative resource allocation strategies, enabling the efficient distribution of computational effort in simulations to achieve a desired level of accuracy with fewer analysis runs^20^.

## Research significance

In this study, the FEMA P-58 framework is adapted and extended through the introduction of a rational building classification scheme and the integration of a Bayesian probabilistic network to establish a systematic and comprehensive approach for urban seismic risk assessment. The proposed methodology is founded on a transparent analytical basis and can be progressively refined as more detailed structural data become available, thereby enhancing the reliability and precision of the results.

In previous research, large-scale seismic risk assessments have predominantly relied on predefined charts and simplified methodologies, while the application of the FEMA P-58 framework in conjunction with Incremental Dynamic Analysis, IDA, for such assessments has not yet been investigated. The principal novelty of this study lies in the integration of this rigorous analytical framework with a Bayesian probabilistic network, thereby establishing a systematic, data-driven, and comprehensive methodology for urban seismic risk evaluation. This approach not only enables the assessment of structural seismic resilience following earthquake events but also facilitates the evaluation of seismic risk across diverse urban areas. The findings of this research provide actionable insights for building insurance companies, municipal authorities, and policymakers, supporting risk-informed decision-making at the strategic level aimed at enhancing urban safety, resilience, and sustainable development. This framework is applied to evaluate the seismic risk of District 2 of Tehran Municipality.

## Data description

The input data used in this study were obtained from multiple authoritative sources and integrated to support the seismic risk assessment framework for District 2 of Tehran. Geographic information, including the spatial distribution of active faults in Tehran, the boundaries of the nine zones of District 2, and the precise location of individual buildings, was acquired in GIS format from the Tehran Disaster Mitigation and Management Organization. In addition, an Excel-based dataset provided by the same organization was used, containing the percentage distribution of each structural type according to the classification defined in this study for the nine zones of District 2 of Tehran Municipality. This dataset, together with the GIS information, formed the core input for large scale seismic risk assessment.

The seismic hazard function for Tehran was adopted from the study conducted by Bazarchi et al.^21^, which provides a region-specific probabilistic seismic hazard model. Damage state probabilities for different performance groups were derived from the Performance Assessment Calculation Tool (PACT) library. These fragility models were used to quantify the relationship between seismic intensity measures and expected damage states for various building categories.

Nonlinear structural response parameters, including Incremental Dynamic Analysis (IDA) results and collapse fragility curves, were obtained through dynamic nonlinear analyses. These results were used to characterize structural performance under increasing seismic intensity levels. Finally, a Bayesian Network framework was employed to integrate the aforementioned datasets and propagate uncertainties through the model. Using the outputs from IDA analyses and the probabilistic relationships defined within the Bayesian network, the probability of damage states for each of the nine zones in District 2 of Tehran was estimated.

To ensure reproducibility of the proposed framework, all major steps of the analysis, including data preprocessing, structural modeling, Incremental Dynamic Analysis (IDA), and Bayesian Network implementation, were conducted using documented and version-controlled scripts. Structural analyses were performed in OpenSees, while the probabilistic modeling and Bayesian inference were implemented using Python-based libraries. Key modeling assumptions, parameter discretizations, and network structures are explicitly described in this study. The complete set of scripts and computational procedures is publicly available in a DOI-minting repository (see Data Availability section), enabling independent replication and further extension of the proposed methodology.

## Bayesian probabilistic network

### Bayesian concept and conditional probability

The term *Bayesian* in this context refers to Bayes’ theorem, attributed to the 18th-century mathematician and philosopher Thomas Bayes, and it forms the basis for updating the probability of an event based on new evidence. In Eq. (1), P(AB) represents the joint probability of events A and B, P(A) is the probability of event A, and P(A∣B) denotes the conditional probability of A given that B has occurred. Bayes’ theorem provides a formal framework for updating event probabilities as new information becomes available. In Eq. (2), the term P(B) is calculated according to the law of total probability^22^. In this equation, $\:\stackrel{-}{A}$ denotes the complement of A.

1 $$\:P\left(A|B\right)=\frac{P\left(AB\right)}{P\left(B\right)}=\frac{P\left(B|A\right)}{P\left(B\right)}P\left(A\right)$$

2 $$\:P\left(B\right)=P\left(BA\right)+P\left(B\stackrel{-}{A}\right)=P\left(B|A\right)P\left(A\right)+P\left(B|\stackrel{-}{A}\right)P\left(\stackrel{-}{A}\right)$$

**Fig. 1:**
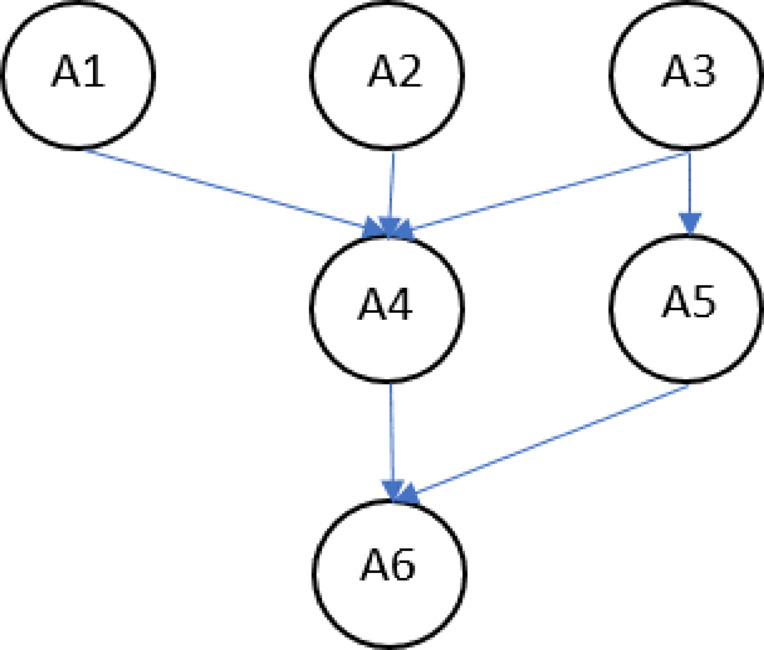
A simplified Bayesian network. (This figure was created by the authors).

A Bayesian probabilistic network consists of a set of parent and child nodes representing random variables and their causal relationships. The conditional dependencies between the nodes are expressed through Conditional Probability Tables (CPTs). Figure 1 illustrates a simple example of a Bayesian network. In Fig. 1, nodes A1, A2, and A3 are defined as parent nodes. Node A4 is the child of all three parent nodes, while node A5 is the child of A3. Both A4 and A5 act as parent nodes for A6. All these conditional dependencies among the nodes are represented using Conditional Probability Tables (CPTs). Probabilistic inference in Bayesian networks (BNs) can be performed in two ways, forward analysis and backward analysis. Forward analysis computes the probability distribution of each node in the BN based on the prior information of the parent nodes and the conditional probability distributions of the nodes. Backward analysis involves determining the probability distribution of parent nodes given observations at one or more child nodes^23^. Therefore, in a Bayesian network, it is possible not only to infer the state of a child node from the conditions and information of its parent node(s), but also, by acquiring information from a given node, to perform backward analysis to evaluate the state of the parent nodes.

### Construction of the Bayesian network

**Fig. 2:**
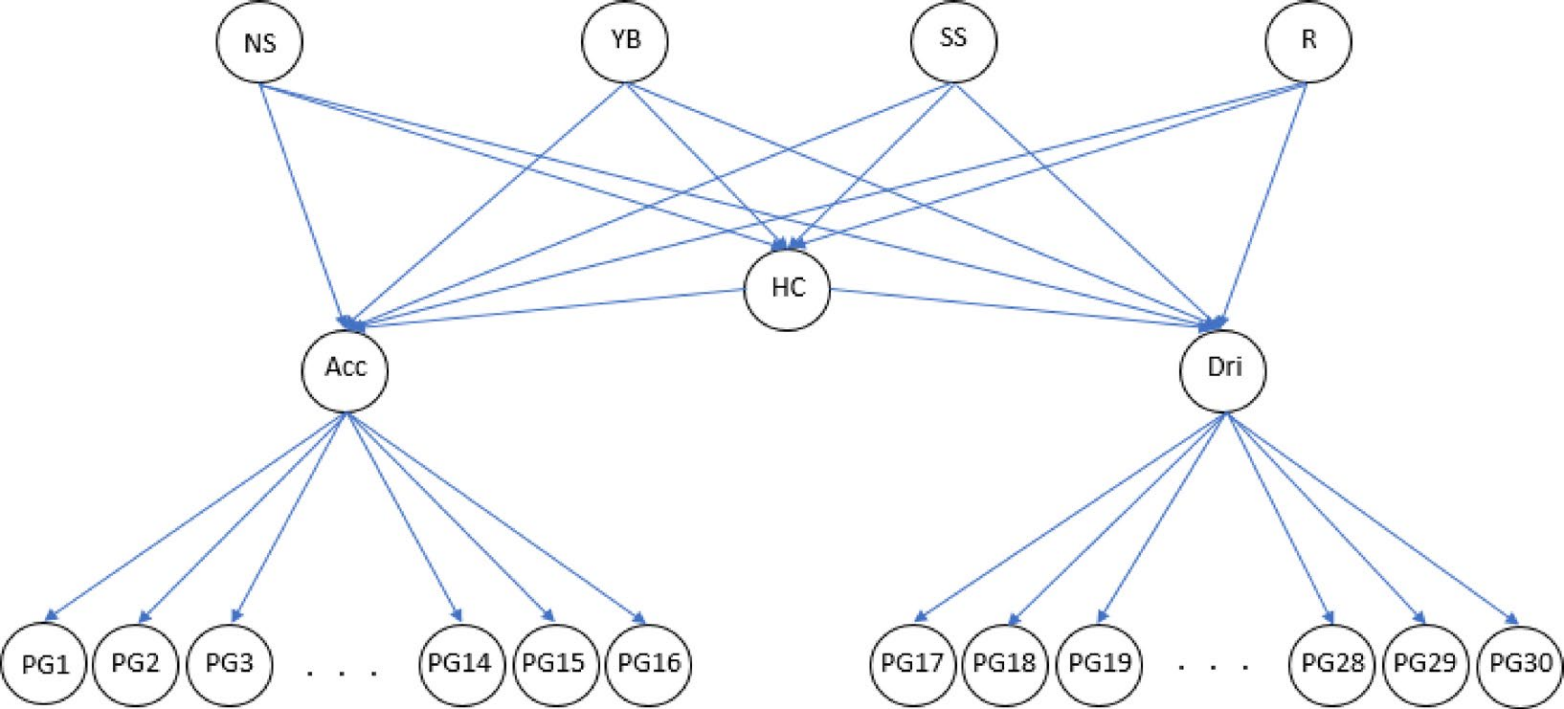
Bayesian probabilistic network for seismic risk assessment. (This figure was created by the authors).

The objective of the Bayesian network in this study is to evaluate urban-scale seismic risk by extending the FEMA P-58 methodology. Accordingly, the nodes defined in the network represent the parameters that influence damage assessment in accordance with this approach. Figure 2 illustrates the Bayesian network employed in this study. Given the large volume of data, the Bayesian probabilistic network was constructed and analyzed in Python using the Bayesian Network and TabularCPD class. Table 1 summarizes the node names, their attributes, possible states, and the conditional probability tables of the parent nodes. The following sections provide detailed descriptions of the nodes and the links connecting them.

- *Node NS* This node represents the probabilistic distribution of buildings based on the number of stories. Two-story buildings represent structures with 1 to 3 stories, five-story buildings represent structures with 4 to 7 stories, and nine-story buildings represent structures with 8 stories or more. These categories were modeled and analyzed accordingly.
- *Node YB* This node represents the construction year of the buildings, categorized according to the edition of the Iranian Seismic Code 2800 in effect. Buildings constructed before 1990 were designed only for gravity loads, buildings constructed between 1990 and 2005 were designed in accordance with the second edition of Code 2800, and buildings constructed after 2005 were modeled and analyzed according to the fourth edition of the Iranian Seismic Code 2800.
- *Node SS* This node represents the structural system of the buildings. Approximately 14% of the buildings in District 2 of Tehran Municipality are masonry, 46% are reinforced concrete, and 40% are steel structures.
- *Node R* This node represents the source-to-site distance. Within the framework of the seismic risk assessment methodology employed in this study, this parameter primarily influences the selection of accelerograms.
- *Node HC* This node represents the seismic hazard curve, which is derived based on regional seismicity, soil type, structural period, and other relevant parameters. It expresses the annual probability of occurrence of different spectral accelerations (Sa).
- *Nodes Acc and Dri* These nodes represent the maximum acceleration and inter-story drift, respectively, obtained from IDA. At this stage, the structures were initially modeled, analyzed, and designed in ETABS according to their number of stories, construction year, and structural system. Table 2 presents the characteristics of the buildings, the design codes, and the structural systems. Tables 3 and 4 provide the dimensions and properties of beams and columns, and the calculated fundamental periods of the steel and reinforced concrete (RC) structures, respectively. Subsequently, considering the source-to-site distance (Node R) in the selection of accelerograms, a total of 81 IDA simulations were performed. The results were then used to construct conditional probability tables for different ranges of acceleration and drift, conditioned on the occurrence of specific spectral acceleration (Sa) intervals, represented by Node HC.
- *Nodes PGi* These nodes represent the characteristics of the performance groups extracted from the Performance Assessment Calculation Tool (PACT) library. Nodes PG1 to PG16 correspond to performance groups that are sensitive to acceleration, while nodes PG17 to PG30 correspond to performance groups that are sensitive to inter-story drift. The list of performance groups is provided in Table 5.

**Table 1:** Bayesian network node specifications.

Node	Name	Type	State	Value
NS	Number of stories	Building specifications	1 ~ 3 (2Stories) 4 ~ 7 (5Stories) 8 & more (9Stories)	0.4213 0.5335 0.0452
YB	Year built	Building specifications	Before 1990 1990 ~ 2005 After 2005	0.2212 0.4581 0.3207
SS	Structural system	Building specifications	Masonry Reinforced Concrete Steel	0.1427 0.4546 0.4027
R	Rupture distance	Seismic Information	*R*<10 km 10 km < *R*<25 km 25 km < *R*<40 km	0.13545 0.64810 0.21645
HC	Hazard curve	Seismic Information	0.5 < Sa < 0.8 0.8 < Sa < 1.15 1.15 < Sa < 1.55 1.55 < Sa < 2.0 2.0 < Sa < 2.5 Sa > 2.5	The hazard curve is selected based on the natural period of the structures.
Acc	Peak floor acceleration	Seismic response parameters	PFA < 0.1 g 0.1 g < PFA<0.2 g 0.2 g < PFA<0.3 g 0.3 g < PFA<0.45 g 0.45 g < PFA<0.6 g 0.6 g < PFA<0.8 g 0.8 g < PFA<1.0 g PFA > 1.0 g	CPD of the Acc node is calculated based on each state of its parent nodes
Dri	Maximum story drift	Seismic response parameters	∆<0.01 0.01<∆<0.02 0.02<∆<0.03 0.03<∆<0.04 0.04<∆<0.05 0.05<∆<0.06 0.06<∆<0.07 ∆>0.07	CPD of the Dri node is calculated based on each state of its parent nodes
PG1 ~ 16	Performance group 1 ~ 16	Damage Ratio (Acceleration Base PGs)	D < 0.1 0.1 < D < 0.25 0.25 < D < 0.5 0.5 < D < 1.0	The amount of each PGi nodes is calculated based on the states of Acc node
PG17 ~ 30	Performance group 17 ~ 30	Damage Ratio (Drift Base PGs)	D < 0.1 0.1 < D < 0.25 0.25 < D < 0.5 0.5 < D < 1.0	The amount of each PGi nodes is calculated based on the states of Dri node

**Table 2:** Structural characterestics and design codes.

Building category	Representative model	Total height (m)	Plan	Seismic design code	Structural system
1–3 Stories – before 1990	2 stories	6.4	3 × 6 m*	Gravity loads only*	Steel IMF
					RC IMRF
4–7 Stories – before 1990	5 stories	16.0	4 × 6 m	Gravity loads only	Steel IMF
					RC IMRF
8 or more Stories – before 1990	9 stories	28.8	5 × 6 m	Gravity loads only	Steel IMF
					RC IMRF
1–3 Stories – 1990–2005	2 stories	6.4	3 × 6 m	Standard 2800–2nd edition	Steel IMF
					RC IMRF
4–7 Stories – 1990–2005	5 stories	16.0	4 × 6 m	Standard 2800–2nd edition	Steel IMF
					RC IMRF
8 or more Stories – 1990–2005	9 stories	28.8	5 × 6 m	Standard 2800–2nd edition	Steel IMF
					RC IMRF
1–3 Stories – after 2005	2 stories	6.4	3 × 6 m	Standard 2800–4th edition	Steel IMF
					RC IMRF
4–7 Stories – after 2005	5 stories	16.0	4 × 6 m	Standard 2800–4th edition	Steel IMF
					RC IMRF
8 or more Stories – after 2005	9 stories	28.8	5 × 6 m	Standard 2800–4th edition	Steel IMF
					RC IMRF

3 × 6 m^*^: Three 6-meter bays in each plan direction Gravity Loads only*: No seismic code.

**Table 3:** Section properties of structural elements and fundamental periods of steel structures.

Building category	Story	Columns Sec.	Beam Sec.(I)	Computational period
S2* – before 1990	1,2	BOX 160 × 160 × 14	PL330 × 8 + 2PL150 × 12	1.095
S2–1990–2005	1,2	BOX 200 × 200 × 16	PL330 × 8 + 2PL150 × 12	0.835
S2 – after 2005	1,2	BOX 240 × 240 × 16	PL330 × 10 + 2PL150 × 15	0.689
S5* – before 1990	1,2	BOX 200 × 200 × 20	PL330 × 8 + 2PL150 × 12	2.154
	3	BOX 180 × 180 × 16	PL330 × 8 + 2PL150 × 12	
	4,5	BOX 160 × 160 × 16	PL330 × 8 + 2PL150 × 12	
S5–1990–2005	1,2	BOX 280 × 280 × 25	PL350 × 10 + 2PL170 × 15	1.476
	3	BOX 240 × 240 × 20	PL350 × 10 + 2PL170 × 15	
	4,5	BOX 220 × 220 × 20	PL330 × 10 + 2PL150 × 15	
S5 – after 2005	1,2	BOX 300 × 300 × 25	PL350 × 12 + 2PL170 × 17	1.365
	3	BOX 260 × 260 × 20	PL350 × 12 + 2PL170 × 17	
	4,5	BOX 240 × 240 × 20	PL330 × 10 + 2PL150 × 15	
S9* – before 1990	1,2,3	BOX 240 × 240 × 25	PL330 × 8 + 2PL150 × 12	3.450
	4,5,6	BOX 200 × 200 × 20	PL330 × 8 + 2PL150 × 12	
	7,8,9	BOX 180 × 180 × 16	PL330 × 8 + 2PL150 × 12	
S9–1990–2005	1,2,3	BOX 340 × 340 × 30	PL380 × 12 + 2PL200 × 18	2.093
	4,5,6	BOX 300 × 300 × 30	PL380 × 12 + 2PL180 × 17	
	7,8,9	BOX 300 × 300 × 20	PL350 × 10 + 2PL170 × 15	
S9 – after 2005	1,2,3	BOX 400 × 400 × 30	PL400 × 15 + 2PL200 × 20	1.836
	4,5,6	BOX 340 × 340 × 30	PL400 × 12 + 2PL200 × 18	
	7,8,9	BOX 300 × 300 × 25	PL350 × 12 + 2PL170 × 17	

S2*: Tow-Story Steel Structure represents 1–3 Stories.

S5*: Five-Story Steel Structure represents 4–7 Stories.

S9*: Nine-Story Steel Structure represents 8 or more Stories.

**Table 4:** Section properties of structural elements and fundamental periods of RC structures.

Building category	Story	Columns Sec.	Beam Sec.	Stirrup details	Computational period
C2 – before 1990	1,2	Cl400 × 400-8Φ14	B400 × 400-6Φ14,4Φ14	Φ10@200	0.666
C2–1990–2005	1,2	Cl450 × 450-12Φ16	B400 × 500-6Φ16,3Φ16*	Φ10@150	0.536
C2 – after 2005	1,2	Cl450 × 450-12Φ18	B400 × 500-5Φ18,3Φ18	Φ10@150	0.515
C5 – before 1990	1,2,3	Cl500 × 500-12Φ16	B400 × 500-4Φ16,3Φ16	Φ10@200	1.341
	4,5	Cl400 × 400-12Φ16	B400 × 500-4Φ16,3Φ16	Φ10@200	
C5–1990–2005	1,2	Cl600 × 600-20Φ18	B400 × 600-6Φ20,4Φ20	Φ12@150	1.050
	3	Cl500 × 500-16Φ18	B400 × 600-6Φ20,4Φ20	Φ12@150	
	4,5	Cl500 × 500-12Φ18	B400 × 500-4Φ20,3Φ20	Φ10@150	
C5 – after 2005	1,2	Cl600 × 600-20Φ20	B400 × 600-6Φ20,4Φ20	Φ12@120	1.050
	3	Cl500 × 500-16Φ20	B400 × 600-6Φ20,4Φ20	Φ12@120	
	4,5	Cl500 × 500-12Φ20	B400 × 500-4Φ20,3Φ20	Φ12@150	
C9 – before 1990	1,2,3	Cl600 × 600-20Φ20	B400 × 500-4Φ16,3Φ16	Φ10@200	2.395
	4,5,6	Cl500 × 500-16Φ20	B400 × 500-4Φ16,3Φ16	Φ10@200	
	7,8,9	Cl500 × 500-12Φ20	B400 × 500-4Φ16,3Φ16	Φ10@200	
C9–1990–2005	1,2,3	Cl600 × 600-28Φ20	B400 × 700-7Φ20,5Φ20	Φ12@150	1.786
	4,5,6	Cl600 × 600-20Φ20	B400 × 600-7Φ20,5Φ20	Φ12@150	
	7,8,9	Cl500 × 500-12Φ20	B400 × 500-5Φ20,3Φ20	Φ10@150	
C9 – after 2005	1,2,3,4	Cl600 × 600-32Φ20	B400 × 700-8Φ20,5Φ20	Φ12@120	1.718
	5,6,7	Cl600 × 600-24Φ20	B400 × 600-7Φ20,5Φ20	Φ12@150	
	8,9	Cl500 × 500-16Φ20	B400 × 500-5Φ20,3Φ20	Φ10@150	

B400 × 500-6Φ16, 3Φ16*: Beam 400 mm (width) × 500 mm (height) reinforced with 6Φ14 (top) and 6Φ14 (bottom).

**Table 5:** Performance groups.

Code	PG name	Type	Acceleration (Acc)/drift	Short description
PG1	E2022.010	Building content	Acc	Unsecured fragile objects on shelves, unknown restraint
PG2	E2022.020	Building content	Acc	Home entertainment equipment, unknown installation
PG3	E2022.023	Building content	Acc	Desktop electronics including computers, monitors, etc.
PG4	B3011.014	Architectural	Acc	Clay tile roof
PG5	B3031.002a	Architectural	Acc	Masonry Chimney - unreinforced
PG6	C3027.001	Architectural	Acc	Raised Access Floor, non seismically rated.
PG7	C3032.003c	Architectural	Acc	Suspended Ceiling
PG8	D1014.021	Mechanical	Acc	Hydraulic Elevator
PG9	D2021.023b	Mechanical	Acc	Cold or Hot Potable Water Piping
PG10	D2022.012a	Mechanical	Acc	Heating hot Water Piping
PG11	D3031.012e	Mechanical	Acc	Chiller - Vibration isolated equipment
PG12	D3041.012a	Mechanical	Acc	HVAC Galvanized Sheet Metal Ducting
PG13	D3052.011b	Mechanical	Acc	Air Handling Unit -Unanchored equipment
PG14	D4011.032a	Mechanical	Acc	Fire Sprinkler Drop Standard Threaded Steel
PG15	D5011.011c	Mechanical	Acc	Transformer/primary service - Unanchored equipment
PG16	D5012.023e	Mechanical	Acc	Low Voltage Switchgear - Vibration isolated
PG17	B2022.038	Architectural	Drift	stick-built curtain wall
PG18	B2023.032	Architectural	Drift	Full tempered Monolithic Storefront
PG19	C3011.001a	Architectural	Drift	Wall Partition, Type: Gypsum + Wallpaper, Full Height
PG20	B1041.021a	Structural	Drift	IMF, Beam one side
PG21	B1041.021b	Structural	Drift	IMF, Beam both sides
PG22	B1044.022	Structural	Drift	concrete walls with double curtain
PG23	B1044.082	Structural	Drift	reinforced concrete walls with boundary columns
PG24	B1049.011	Structural	Drift	Reinforced concrete flat slabs- columns
PG25	C2011.011b	Structural	Drift	concrete stair with no seismic joint
PG26	B1031.011a	Structural	Drift	Steel Column Base Plates
PG27	B1031.021a	Structural	Drift	Welded column splices
PG28	B1035.001	Structural	Drift	Post-Northridge RBS connection, beam one side of column only
PG29	B1035.011	Structural	Drift	Post-Northridge RBS connection, beams both sides of column
PG30	C2011.001b	Structural	Drift	steel stair without seismic joint

## Seismic risk assessment

As previously noted, the seismic risk assessment in this study is conducted using an enhanced implementation of the FEMA P-58 methodology^12^.

This framework is fundamentally based on the following conditional integral formulation. In this formulation, DV denotes the *Decision Variable*; DM refers to the *Damage Measure*; EDP indicates the *Engineering Demand Parameter*; IM corresponds to the *Intensity Measure*; and f(IM) represents the probability density function of the selected intensity measure. The overall procedure adopted in this research is summarized in the following steps.

3 $$\:F\left(DV\right)=\iiint\:F\left(DV|DM\right)df\left(DM|EDP\right)df\left(EDP|IM\right)f\left(IM\right)dIM$$

Step 1: $$\:f\left(IM\right)dIM$$

This stage represents the probabilistic characterization of seismic intensity, derived from the seismic hazard curve, and corresponds to the HC node in the Bayesian network. The seismic hazard function for Tehran was adopted from the study conducted by Bazarchi et al.^21^ The fundamental period of each structural typology was first calculated, and the corresponding hazard curve, associated with this fundamental period, was obtained through interpolation. To improve the relevance and robustness of the analysis, only spectral acceleration values exceeding 0.5 g were considered. Consequently, the Conditional Probability Distribution (CPD) of the HC node represents the conditional probability of occurrence of different Sa intervals, given that the ground motion intensity exceeds 0.5 g, rather than the annual exceedance probability of Sa.

Step 2: $$\:df\left(EDP|IM\right)$$

In this stage, IDA was performed to derive the seismic demand parameters, with the maximum inter-story drift ratio and peak floor acceleration considered as the primary engineering demand parameters governing structural and non-structural damage. The initial stage of the IDA procedure involves selecting ground motion records consistent with the expected source-to-site rupture distance. For near-field conditions, 14 pairs of ground motion records recommended by PEER were employed. For far-field conditions, a combination of PEER ground motions and additional accelerograms recorded at stations located at distances compatible with the target source-to-site rupture distance was utilized. Specifically, 22 pairs of records were used for source-to-site rupture distances of 15–25 km, and 14 pairs for distances of 25–40 km. A complete list of the selected ground motion records is provided in Table 6.

**Table 6:** Ground motion specifications.

Record Name	Mw	Year	Mechanism	Station name
Imperial Valley	6.53	1997	Strike-slip	El Centro Array #6, #7, #11, Delta, Plaster City
Irpinia Italy	6.9	1980	Normal–oblique	Sturno (STN),
Superstition Hills	6.54	1987	Strike-slip	Parachute Test Site, El Centro IMP Co Center, POE, Calipatria Fire Station
Loma Prieta	6.93	1989	Reverse-oblique	Saratoga - Aloha Ave, Capitola, Gilroy Array, Halls Valley
Erzican	6.7	1992	Strike-slip	Erzincan
Cape Mendocino	7.01	1992	Reverse	Petrolia, Rio Dell Overpass, College of the Redwoods
Landers	7.28	1992	Strike-slip	Lucerne, Yermo Fire Station, Coolwater, Whitewater Trout Farm
Northridge	6.69	1994	Reverse	Rinaldi Receiving Sta, Sylmar - Olive View Med FF, Beverly Hills – 14,145, Canyon Country, LA - Baldwin Hills
Kocaeli	7.51	1999	Strike-slip	Izmit, Duzce, Arcelik, Iznik
Chi-Chi	7.62	1999	Reverse-oblique	TCU065, TCU102, CHY101, TCU045, CHY087
Duzce	7.14	1999	Strike-slip	Duzce, Bolu, Mudurnu
Kobe	6.9	1995	Strike-slip	Nishi-Akashi, Shin-Osaka, Tadoka
San Fernando	6.61	1971	Reverse	La Hollywood Stor Lot, Pearblossom Pump
Friuli	6.5	1976	Reverse	Tolmezzo, Codroipo
Manjil	7.37	1990	Strike-slip	Manjil, Qazvin
Hector Mine	7.13	1999	Strike-slip	HEC

The active faults influencing seismic hazard in the study area include the North Tehran Fault, the North Rey Fault, and the South Rey Fault. The probability associated with each source-to-site rupture distance scenario (node R) was computed based on the spatial position of Tehran’s District 2 relative to these faults, assuming a uniform likelihood of seismic activation along the fault length.

### Incremental dynamic analysis (IDA)

To account for the full range of structural member behavior prior to global collapse, the nonlinear behavior of structural materials must be explicitly considered. Nonlinear material modeling is generally performed using either the concentrated plasticity or distributed plasticity formulation^24^. In nonlinear structural analysis adopting the concentrated plasticity approach, as implemented in this study, the inelastic behavior is assumed to be localized at discrete regions along the members. The nonlinear response of structural elements was represented by assigning rotational spring elements at beam ends and column bases, incorporating the Bilin-Materials constitutive model. This model was originally introduced by Ibarra, Medina, and Krawinkler^25^ and later refined by Lignos and Krawinkler^26^ to account for parameters such as asymmetric cyclic deterioration and residual strength. To incorporate the inherent uncertainties in structural response derived from IDA, each ground motion record was appropriately scaled to cover a wide range of seismic intensities. Studies by Shome and Cornell^27^ demonstrated that the first-mode spectral acceleration serves as a suitable intensity measure for moment-resisting frame systems. Accordingly, in this study, the first-mode spectral acceleration was adopted as the intensity measure. Each ground motion record was initially scaled to multiple intensity levels, and dynamic analyses were then performed for each structural typology under the effects of the scaled records at varying intensities. To provide a comprehensive representation of structural behavior, the Hunt & Fill algorithm was employed^28^, and the criteria for determining the global collapse point in the IDA were applied as described below.

- Occurrence of a slope equal to 20% of the initial elastic slope in the Incremental Dynamic Analysis (IDA) curve.Exceeding a maximum inter-story drift ratio of 0.1.
- Numerical divergence of the structural analysis algorithm.

**Fig. 3:**
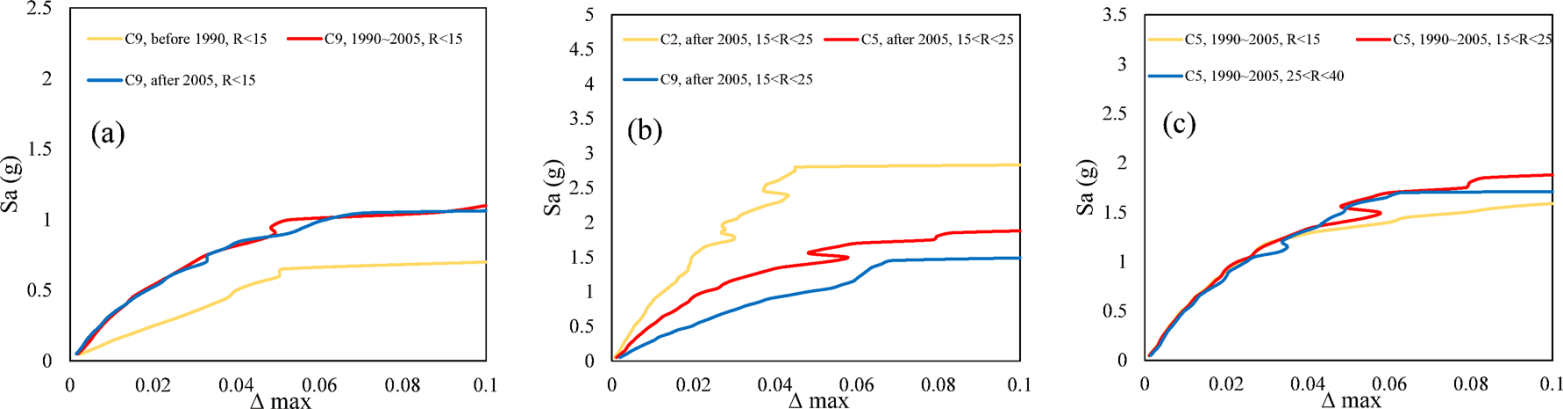
Median IDA curves for reinforced concrete structures: (**a**) year built, (**b**) number of stories, and (**c**) source-to-site distance. (This figure was generated by the authors using the scripts provided in the public repository (DOI: 10.5281/zenodo.19560389)).

**Fig. 4:**
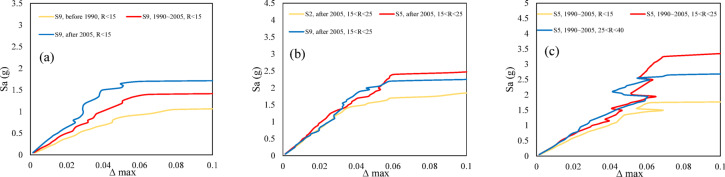
Median IDA curves for steel structures: (**a**) year built, (**b**) number of stories, and (**c**) source-to-site distance. (This figure was generated by the authors using the scripts provided in the public repository (DOI: 10.5281/zenodo.19560389)).

**Fig. 5:**
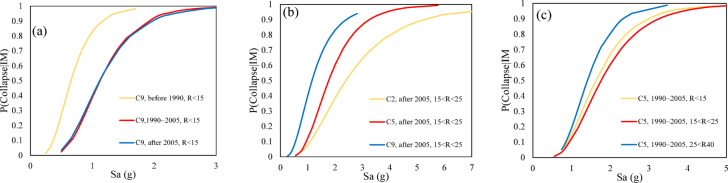
Collapse curves for reinforced concrete structures: (**a**) year built, (**b**) number of stories, and (**c**) source-to-site distance. (This figure was generated by the authors using the scripts provided in the public repository (DOI: 10.5281/zenodo.19560389)).

**Fig. 6:**
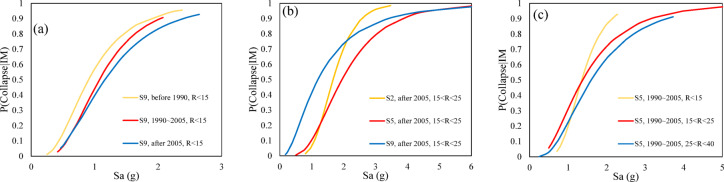
Collapse curves for steel structures: (**a**) year built, (**b**) number of stories, and (**c**) source-to-site distance. (This figure was generated by the authors using the scripts provided in the public repository (DOI: 10.5281/zenodo.19560389)).

Figures 3 and 4 present the IDA 50% curve (i.e., the median IDA curve) for reinforced concrete and steel structures, respectively, and Figs. 5 and 6 illustrate the Collapse curves for reinforced concrete and steel structures, respectively, which are used to compare and assess the performance of different structural typologies and to illustrate the effects of parameters such as year built, number of stories, structural system, and source-to-site distance. The following observations can be drawn from the comparison of these curves.

- New structures, benefiting from the more stringent provisions of contemporary seismic codes, typically exhibit enhanced earthquake performance, with reduced inter-story drifts under a given ground motion intensity and a lower probability of collapse.
- In general, the maximum inter-story drift ratio in steel structures is lower than in concrete structures, with this distinction being more pronounced for high-rise buildings. Assuming all other conditions are equal, the maximum drift and probability of collapse in a 9-story concrete building are significantly higher than in a 2-story concrete building, whereas no substantial difference is observed for steel structures.
- Overall, near-field earthquakes induce larger inter-story drifts and higher probabilities of collapse compared to far-field events, with this effect being more pronounced in steel structures.

**Fig. 7:**
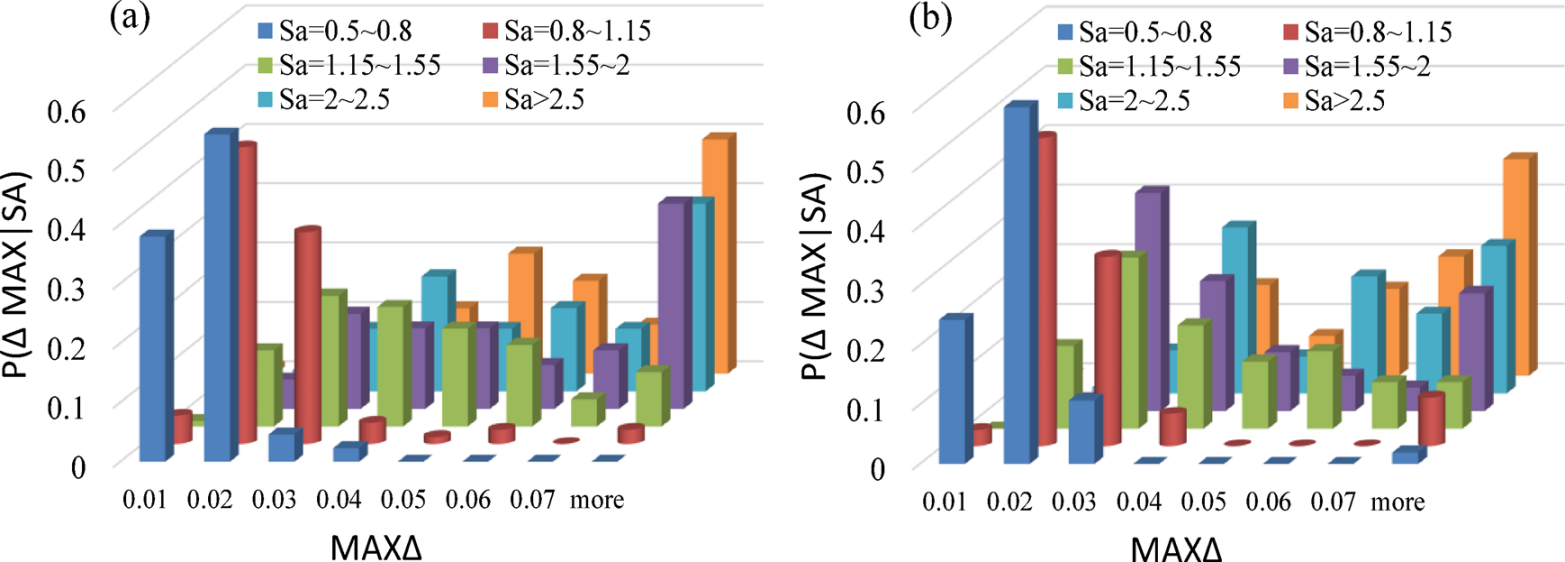
Probability distribution of maximum drift values for 5-story buildings constructed after 2005 with 15 < *R* < 25: (**a**) reinforced concrete, (**b**) steel structure. (This figure was generated by the authors using the scripts provided in the public repository (DOI: 10.5281/zenodo.19560389)).

**Fig. 8:**
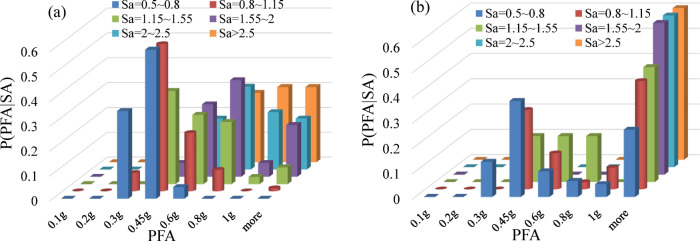
Probability distribution of peak floor acceleration values for 5-story buildings constructed after 2005 with 15 < *R* < 25: (**a**) reinforced concrete, (**b**) steel structure. (This figure was generated by the authors using the scripts provided in the public repository (DOI: 10.5281/zenodo.19560389)).

By performing IDA and extracting the maximum inter-story drift ratio and peak floor acceleration at different spectral acceleration (Sa) levels, the conditional probability tables for the drift and acceleration nodes are obtained. These tables represent the conditional probability of occurrence of various drift and acceleration ranges given different Sa intervals, for different structural typologies and source-to-site distances. The graphs presented in Figs. 7 and 8 serve as examples of these conditional probability tables, illustrating these two parameters for a 5-story concrete and steel building constructed after 2005, with a rupture distance of 15 km < *R* < 25 km. Overall, it can be observed that, under identical conditions, the maximum drift is higher in the concrete structure, whereas the maximum acceleration is higher in the steel structure.

Step 3: $$\:df\left(DM|EDP\right)$$

**Fig. 9:**
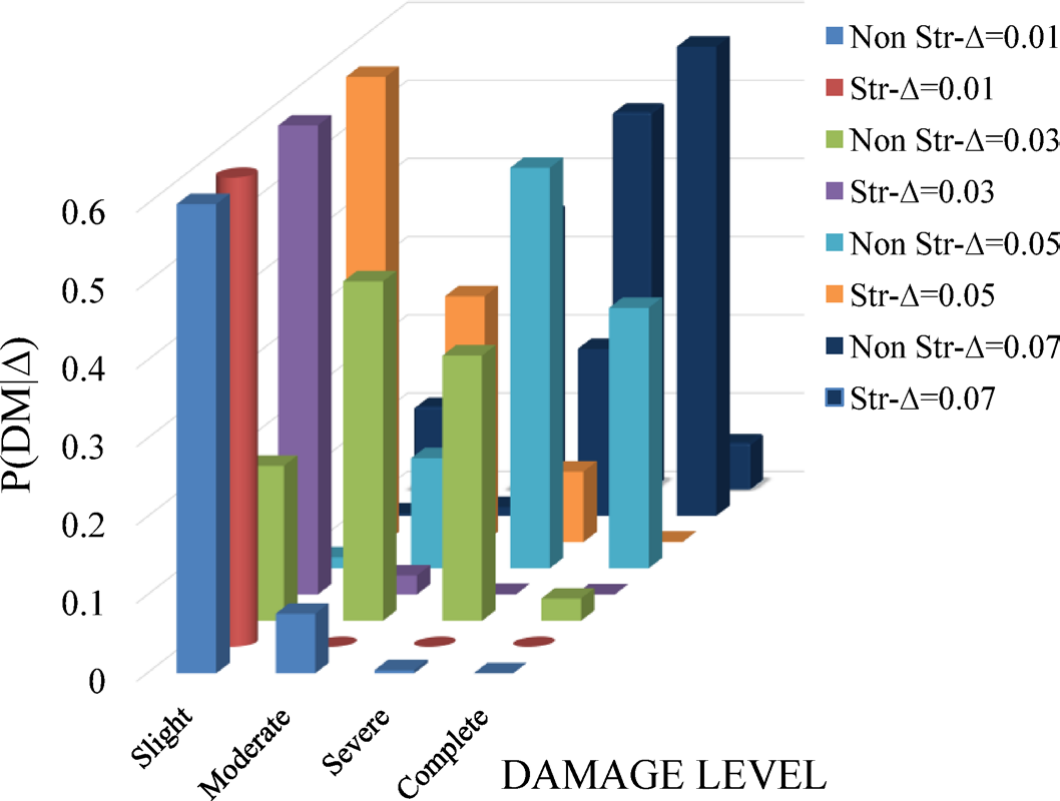
Comparison of the probabilistic distribution of damage levels for drift-sensitive structural and non-structural components. (This figure was generated by the authors using the scripts provided in the public repository (DOI: 10.5281/zenodo.19560389)).

The third stage of the proposed methodology focuses on estimating the percentage of damage based on the calculated seismic demand parameters. To this end, the PACT library provides a comprehensive database derived from past earthquake records and experimental studies of structural elements, non-structural components, and building contents. The elements are classified into acceleration-sensitive and drift-sensitive categories. By integrating this library with Monte Carlo simulations, the conditional probabilities of various damage levels, given different drift and acceleration intervals, were evaluated for 16 acceleration-sensitive and 14 drift-sensitive performance groups. In total, 64,000 Monte Carlo simulations were conducted for each performance group, resulting in the conditional probability distribution tables for the PGi nodes. The graphs in Fig. 9 illustrate the conditional probability tables for the two performance groups B1031.011a and C3011.001a, representing a structural and a non-structural element, respectively, both of which are drift-sensitive. From these graphs, it is evident that the non-structural element experiences significantly higher damage than the structural element under the same drift level.

## Bayesian network analysis and results

The initial dataset for this study was obtained from the Tehran Emergency Management and Disaster Prevention Organization. District 2 of Tehran Municipality, covering an area of 47.1 km², is subdivided into nine urban zones. Bayesian analyses were performed independently for each zone, utilizing the available data on the number of buildings corresponding to each structural typology. The conditional probability tables (CPTs) of the PGi nodes, representing the output of the Bayesian network, were generated through forward analysis. Subsequent Bayesian evaluation provided the conditional probabilities of varying damage levels for each performance group within the different structural typologies. By separately averaging the results for drift-sensitive and acceleration-sensitive performance groups, the probability distributions of damage levels for each building type were obtained.

**Fig. 10:**
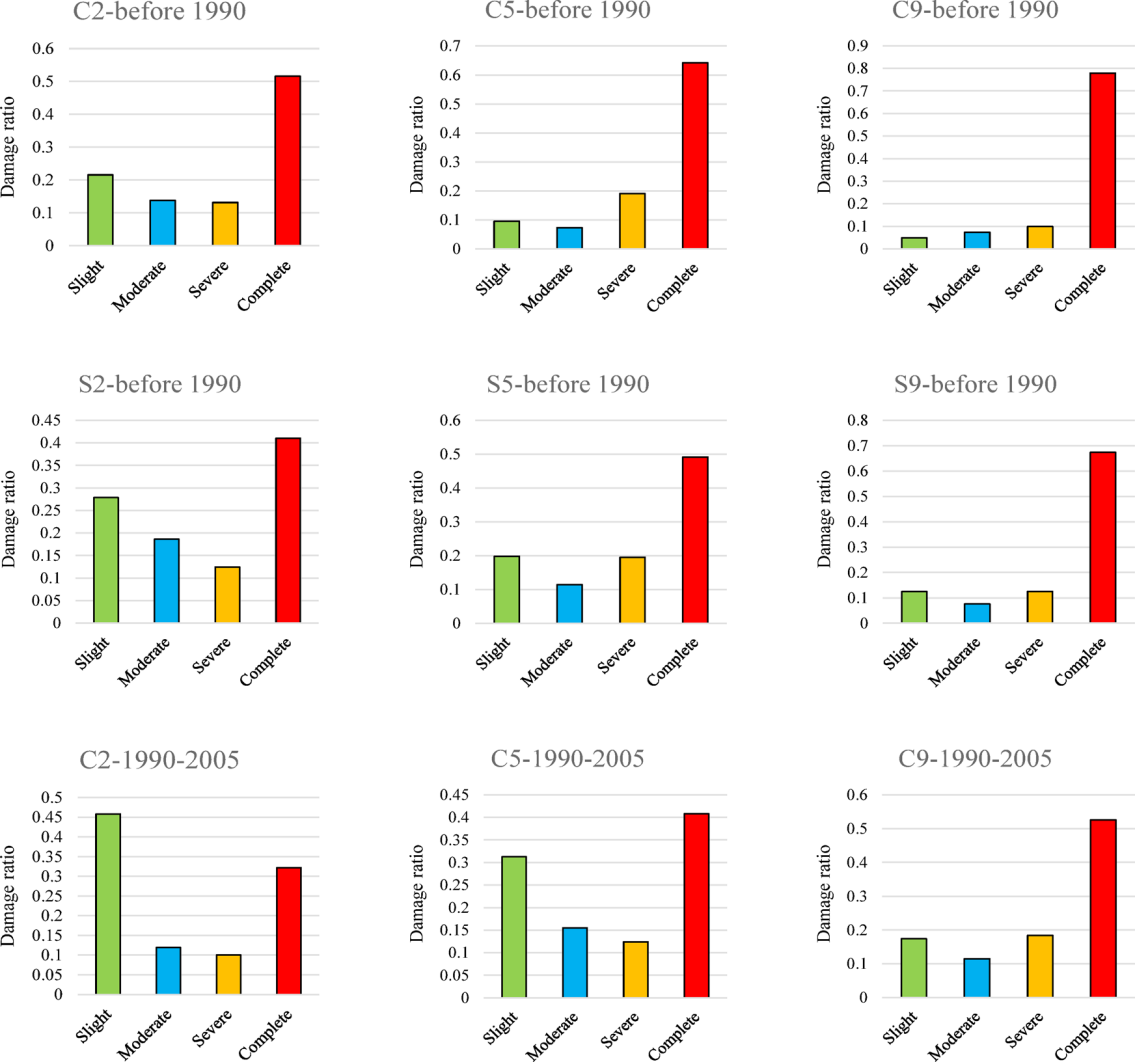

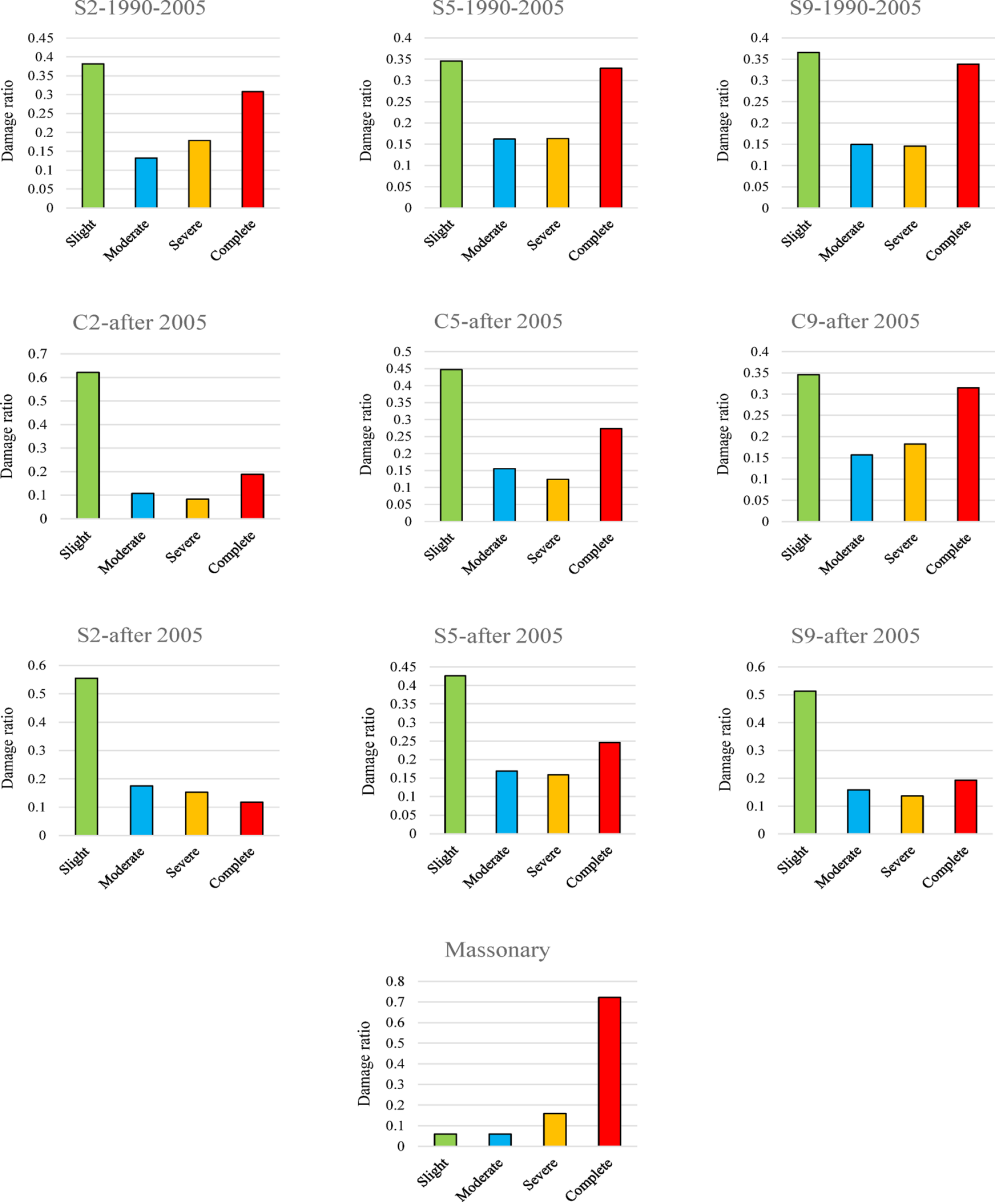
Probabilistic distribution of damage levels for different structures. (This figure was generated by the authors using the scripts provided in the public repository (DOI: 10.5281/zenodo.19560389)).

As presented in Table 1, four damage levels were considered, ranging from slight to complete damage, corresponding to Slight Damage, Moderate Damage, Severe Damage, and Complete Damage, respectively. Damage exceeding 50% was classified as complete damage, as such a level of destruction typically necessitates reconstruction. Figure 10 illustrates the resulting damage probability distributions for various structural typologies. These distributions represent the likelihood of different damage states occurring given a seismic event with Sa > 0.5 g, with damage quantified in terms of the percentage of reconstruction cost relative to the original building.

**Fig. 11:**
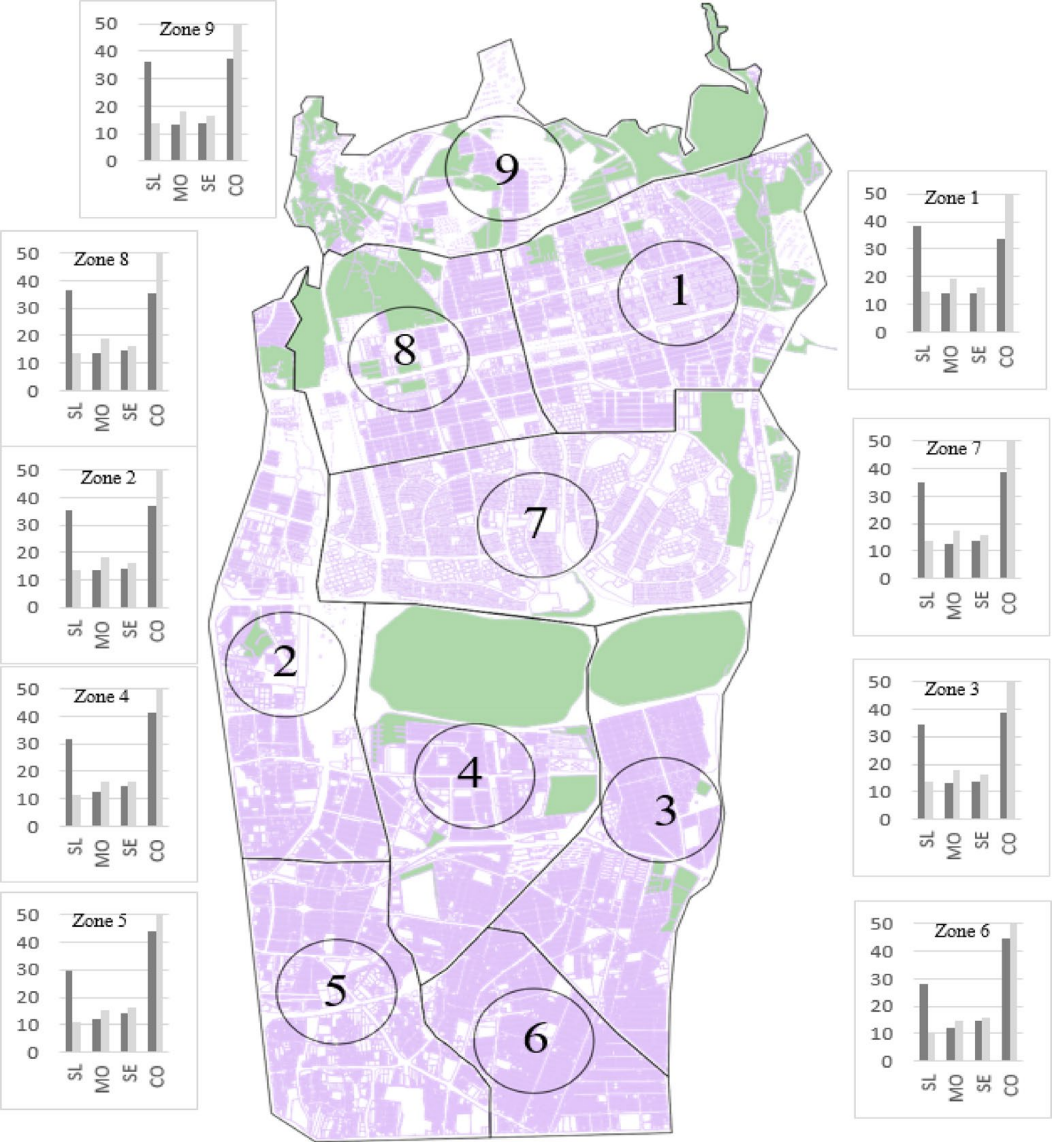
Nine zones of District 2 in Tehran with probabilistic damage distribution for acceleration-sensitive (dark grey shade) and drift-sensitive (light grey shade) components of each zone. (The geographic zoning is based on GIS data from the Tehran Municipality Crisis Management Organization. The probabilistic damage distribution bar charts was generated by the authors using the scripts provided in the public repository (DOI: 10.5281/zenodo.19560389)).

By combining the proportion of each structural typology within each urban zone of District 2 of Tehran with the conditional probabilities of damage for both drift-sensitive and acceleration-sensitive components, zone-specific probability distributions of total damage were derived. The resulting damage maps, overlaid with the zoning of District 2, are presented in Fig. 11.

## Limitations

Despite the robustness of the proposed seismic risk assessment framework, several limitations related to data availability, modeling assumptions, and computational constraints should be acknowledged. A primary limitation arises from the datasets provided by the Tehran Disaster Mitigation and Management Organization. The GIS dataset obtained from this organization did not include key building attributes such as construction year, number of stories, structural system, or occupancy information. As a result, an additional Excel-based dataset was used, which only provided the percentage distribution of structural types according to the classification defined in this study across the nine subzones of District 2. This constraint limited the level of detail available for building-specific vulnerability characterization and introduced a degree of aggregation uncertainty in the exposure model.

Furthermore, the study is restricted to District 2 of Tehran due to data availability, which limits the generalizability of the results to other urban regions. Although the seismic hazard model adopted from Bazarchi et al.^21^ provides a region-specific probabilistic representation for Tehran, epistemic uncertainties in hazard estimation and ground motion characterization may still influence the overall risk outputs. These uncertainties are partially propagated through the Bayesian Network framework but cannot be entirely eliminated.

Due to the large spatial scale of the study and the associated computational demands, simplifying assumptions were introduced in both the Incremental Dynamic Analysis (IDA) and the Bayesian Network modeling. In particular, nonlinear structural analyses in OpenSees were performed using two-dimensional frame models instead of full three-dimensional representations. While this simplification introduces approximation error, it significantly reduces computational cost and enables large-scale probabilistic analysis. Moreover, the Bayesian Network framework does not explicitly include some potentially influential variables, such as liquefaction potential, and building occupancy characteristics.

Finally, the accuracy of the proposed framework depends on the structure of the Bayesian Network and the assumed conditional dependencies between variables. Although the model captures key interactions between hazard, fragility, and damage, necessary simplifications for computational tractability may limit its ability to fully represent complex real-world system behavior.

## Conclusions

In this study, a framework for large-scale seismic risk assessment was developed that does not rely on predefined fragility curves or overly simplified modeling approaches. Instead, by enhancing the FEMA P-58 methodology and incorporating Incremental Dynamic Analysis (IDA) alongside a Bayesian probabilistic network, the proposed approach enables a systematic assessment of seismic risk a large scale. Through an appropriate classification of buildings based on construction year, number of stories, and structural system, this approach enables not only the prediction of the seismic performance of individual structures, but also the identification of urban areas with higher vulnerability to earthquake hazards. The results of this assessment can be used to support strategic retrofit planning and to inform disaster risk management policies.

The results indicated that the percentage of structural damage tends to decrease with the reduction of building age. Moreover, damage to drift-sensitive performance groups appears to be more significant in reinforced concrete structures, while acceleration-sensitive performance groups show relatively higher damage in steel structures. It is also observed that, particularly in reinforced concrete structures, the extent of damage may increase with building height. Furthermore, for earthquake scenarios with Sa > 0.5 g, Zones 5 and 6 in District 2 of Tehran are expected to experience the highest level of complete damage. The proportion of complete damage in these two zones was estimated to be approximately 44% for drift-sensitive elements and 59% for acceleration-sensitive elements.

Access to more comprehensive and detailed primary data, such as accurate structural characteristics of all buildings or more granular building classification information, would further enhance the reliability and precision of the results. Moreover, employing the Bayesian probabilistic network allows the model to be updated as new information becomes available. This includes forward or backward inference adjustments when new data are obtained from any node in the network, such as building-specific properties or observed earthquake damage. Consequently, the probabilities associated with other nodes may be revised, potentially improving the adaptability and accuracy of the seismic risk assessment framework.

## Data Availability

The datasets generated and/or analyzed during the current study are available from the corresponding author Mehdi Banazade (mbanazadeh@aut.ac.ir), upon reasonable request.In addition, all codes, scripts, and processed datasets supporting the findings of this study have been publicly archived in a DOI-minting repository. The repository is accessible at: https://doi.org/10.5281/zenodo.19560389The repository includes: Data preprocessing scripts, Bayesian Network modeling scripts, Seismic risk assessment workflow, Sample input and output datasets.All data sources used in this study are described in detail within the manuscript, along with their respective access links where applicable.
